# Rationale and efficacy of interleukin-1 targeting in pediatric Erdheim- Chester disease

**DOI:** 10.1186/1546-0096-9-S1-P67

**Published:** 2011-09-14

**Authors:** TA Tran, JC Lecron, D Pariente, I Jéru, A Delwail, I Kone-Paut, U Meinzer

**Affiliations:** 1 Department of paediatrics, pediatric Rheumatology. CEREMAI Bicêtre Hospital, university of Paris sud, France; 2 Department of Immunology and Inflammation. Poitiers Hospital, France; 3 Department of pediatric radiology. Bicêtre Hospital, France; 4 Department of Genetics. Trousseau Hospital. Paris. France

## Introduction

Erdheim-Chester disease (ECD) is a rare non-Langerhans systemic histiocytosis of unknown origin. ECD is characterized by bilateral and symmetric sclerosis of the metaphyseal regions of the long bones and infiltration in other organs. Histopathologically, ECD is characterized by a mononuclear infiltrate of foamy histiocytes.

## Aim of the study

To assess the role of pro-inflammatory cytokines in the physiopathology of the disease and efficacy of IL-1 blockade in treatment of pediatric ECD.

## Patient

A 10 year-old girl with visceral and bone involvement after failure of IFN-α treatment.

## Methods

PBMC were isolated by Ficoll-Paque from whole blood, cultured in RPMI and stimulated with LPS. IL-1, 6 and TNF-α were measured in supernatant and in blood by ELISA. Patient’s data were compared with 15 healthy controls.

## Result

The patient was followed for three years for daily fever 40°C, hepato-splenomegaly, bone pain of the lower limbs and growth retardation. ESR (105mm/h) and C-reactive protein level (CRP=97mg/l) were high. Bone X ray found multiple osteolytic and osteosclerotic lesions in femurs, tibia, and pelvis. Whole-body MRI showed hyperintensity of the skeletal bone marrow in T2-weighted images and retroperitoneal infiltration. Bone biopsies confirmed ECD. The patient received 3x10^6^ units of IFN-α2a thrice weekly. All symptoms disappeared, ESR and CRP normalized after 4 months. The whole-body MRI showed regression of retroperitoneal infiltration, and improvement of bone marrow intensity. At month 10, the patient experienced a relapse. Serum IL-6 drastically increased 174 pg/ml (N: 0-8.6) but not IL-1 and TNF-α. The patient’s IL-6, IL-1β and TNF-α measurement from supernatant of PBMC culture showed respectively increased levels compared to control, before (71.8ng/ml, **1290p**g/ml, 980pg/ml vs 3±1.1, 32±8.9, 174±99) and after stimulation (155.9ng/ml, **3800**pg/ml, 1250pg/ml vs 90±11, 3770±506, 1433±196). The patient was then treated with Anakinra, an IL-1 receptor antagonist at 2mg/kg/day. In one week, fever, bone pain resolved, ESR and CRP normalized. The child gained 8 kg weight and 6 cm height in 10-month treatment. MRI at month 7 showed stability of bone lesion and retroperitoneal infiltration. No side effect was notified.

## Discussion

Patient’s PBMC secreted high amount of IL1 and IL-6 at basal level which increased notably afer stimulation. This may participate in the patho-physiology of ECD.

## Conclusion

Our study supports a central role of the IL-1 network, which seemed to be overstimulated. Its specific blockade could be safely used in pediatric ECD. But we cannot confirm that this could cure this severe disease.

